# Stroke Incidence and Smoking in American Indians: An Update from the Strong Heart Study

**DOI:** 10.3390/jcm15020431

**Published:** 2026-01-06

**Authors:** Taylor Niznik, Jessica A. Reese, Jason F. Deen, Tauqeer Ali, Amanda M. Fretts, Jason G. Umans, Ying Zhang, Christopher S. Graffeo

**Affiliations:** 1 Department of Neurosurgery, University of Oklahoma Health Sciences Center, Oklahoma City, OK 73104, USA; taylor.niznik@gmail.com; 2 Center for American Indian Health Research, University of Oklahoma Health Sciences Center, Oklahoma City, OK 73104, USAtauqeer-ali@ou.edu (T.A.);; 3 Center for Indigenous Health, Departments of Pediatrics and Medicine, University of Washington, Seattle, WA 98195, USA; 4 Department of Epidemiology, School of Public Health, University of Washington, Seattle, WA 98195, USA; amfretts@u.washington.edu; 5 Georgetown-Howard Universities Center for Clinical and Translational Science, Washington, DC 20057, USA; jgu@georgetown.edu

**Keywords:** stroke, American Indians, smoking, epidemiology, risk factors

## Abstract

**Background/Objectives**: To investigate the relationship between cigarette smoking and long-term stroke outcomes in American Indian participants of the Strong Heart Study (SHS). **Methods**: SHS is a longitudinal, population-based cohort study of cardiovascular disease in American Indian tribes and communities in Oklahoma, Arizona, and the Dakotas. Data were abstracted from 5802 participants without prevalent stroke, enrolled during two asynchronous sampling periods (1989–1991; 2001–2003), who underwent annual surveillance through 31 December 2021. Age- and sex-specific person–time incidence rates of stroke and their 95% CIs were calculated for each cohort. A combined analysis using shared frailty Cox proportional hazards models assessed the association of incident stroke with baseline smoking status, demographics, and other key risk factors. **Results**: Among participants, baseline smoking status was positive in 2220 (38.3%). Incident stroke was observed in 456 (7.9%) during a pooled median follow-up time of 19.54 years (range, 0.02–32.62) across the combined cohorts. Stroke incidence was higher among the original cohort, smokers, and older individuals. Across both cohorts, baseline current smokers had a 2.23-fold higher risk of incident stroke compared to nonsmokers (HR = 2.23, 95%CI = 1.73–2.88) and a 1.69-fold higher risk compared to former smokers (HR = 1.69, 95%CI = 1.34–2.13) after controlling for covariates. **Conclusions**: Smoking remains a key risk factor for stroke in American Indians. The current study extends our prior research with broader age ranges and longer follow-up in two cohorts. While American Indians have higher smoking prevalence and stroke risk, increased risk in current versus former smokers supports smoking cessation benefits and emphasizes the need for culturally tailored interventions.

## 1. Introduction

Stroke is a leading cause of death and long-term adult disability [1]. Risk is heavily modulated by non-modifiable factors such as older age [1], female sex [2,3], and genetic predisposition [4,5], as well as modifiable factors including smoking [6,7,8], hypertension [9,10], hyperglycemia [11,12], dyslipidemia [13,14], alcohol consumption [15,16], physical inactivity and poor diet [17,18]. Despite the increasing stroke risk factor burden, stroke mortality has declined [19,20], in part due to effective primary prevention via hypertension control and smoking cessation programs [21,22]. According to the 2020 National Health Interview Survey (NHIS), the prevalence of cigarette use has declined to 12.5% from 30.6% in 1980 [23,24]; however, it remains high among American Indians as compared to other racial/ethnic groups, at 27.1% [24].

Between 2005–2012, American Indians had the highest prevalence of cigarette smoking among all racial/ethnic groups [25], which may contribute to the population’s disproportionate rates of smoking-related diseases, including stroke [26]. Despite decreasing stroke rates in the American Indian population, rates are still higher than those observed in the general population [27,28]. The 2016 Behavioral Risk Factor Surveillance System (BRFSS) data reported prevalent stroke in 2.7% of NH whites, 4.1% of NH blacks, and 5.3% of American Indian/Alaska Natives [29]. Further analysis is required to characterize how stroke decline in this population parallels concomitant national trends, as well as the degree to which this decline may be attributable to changes in modifiable stroke risk factors. The current study examines the influence of smoking on trends in stroke incidence and risk within a subset of American Indian populations using longitudinal data from two cohorts enrolled in the Strong Heart Study (SHS) during 1989–2021.

## 2. Methods

Due to privacy agreements with participating tribal communities, access to study data is restricted. Qualified researchers with human subject confidentiality training can request dataset access through the SHS Coordinating Center (Oklahoma City, OK, USA) at https://strongheartstudy.org/ (accessed on 1 December 2025).

### 2.1. Study Populations

The SHS is a longitudinal, population-based cohort study of cardiovascular disease and its risk factors in American Indians, covering three geographic areas across Oklahoma, Arizona, and the Dakotas [30]. Participants forming the original cohort were enrolled between 1989–1991, with ages ranging from 45–74 years [30]. The SHS included three clinical examinations of the original cohort: Phase I from 1989–1991, followed by Phase II, and Phase III from 1993–1995 and 1998–1999, respectively. Following Phase III, SHS conducted a Phase IV examination, which included certain original cohort members, those cohort members’ families, and additional families from the same regions (Strong Heart Family Study, 2001–2003) with ages ranging from 14–93 years [31]. All salient study procedures were approved by both our institutional review board and tribal review boards, and participants provided written informed consent for SHS participation [32].

### 2.2. Measures

#### 2.2.1. Baseline Variables

We conducted a retrospective review of the following data. Data collection involved comprehensive medical history, lifestyle questionnaires, a clinical examination, and laboratory assessments [30]. At baseline (Phase I for the original cohort; Phase IV for the family cohort), participants were asked (1) “Have you smoked at least 100 cigarettes in your entire life?” (yes/no), (2) “How old were you when you first started smoking cigarettes fairly regularly?” and (3) “Do you smoke cigarettes now?” (yes/no). Participants were classified as current smokers, former smokers, or nonsmokers. We categorized those who smoked at least 100 lifetime cigarettes and reported currently smoking cigarettes as current smokers, those who smoked at least 100 lifetime cigarettes and reported not currently smoking cigarettes as former smokers, and those who smoked fewer than 100 lifetime cigarettes as nonsmokers [33]. Additional baseline data on smoking included duration in years, daily hours exposed to secondhand smoke, daily cigarette count, and packs per year. While the smoking-related variables used to classify baseline smoking status were available for the entire analytic sample, some of the additional smoking exposure measures had missing data; therefore, descriptive analyses of these variables were restricted to participants with non-missing data for the specific measure of interest, and the corresponding sample sizes are reported in the Results for context.

We collected potential confounding or effect-modifying variables using questionnaires, a physical exam at baseline consisting of anthropometric measurements with participants wearing light clothing, and laboratory tests at baseline [34]. Participants reported sex [male/female] and date of birth, which was used to calculate age at examination and categorize patients into three age groups [<55, 55–64, 65+]. We defined current alcohol use as those who reported any alcohol consumption within the past year [35]. We used the American Diabetes Association guidelines to define diabetes mellitus, which included a fasting plasma glucose ≥ 7.0 mmol/L [126 mg/dL] or use of diabetes medication [36]. We measured sitting blood pressure three consecutive times following a 5 min rest using mercury sphygmomanometers and used the average of the second and third systolic and diastolic blood pressure readings in the analysis [37]. Current hypertension was defined according to criteria outlined in the Joint National Committee on Prevention, Detection, Evaluation, and Treatment of Hypertension (JNC-7) [38]: (1) taking an antihypertensive drug, (2) systolic blood pressure ≥ 140 mmHg, or (3) diastolic blood pressure ≥ 90 mmHg. Micro- and macroalbuminuria were determined via urine albumin/creatinine ratios of 30–299 mg/g and ≥300 mg/g, respectively. Baseline HDL-C levels were categorized into three groups: ≥60 mg/dL (high), 40–59 mg/dL (intermediate), and <40 mg/dL (low) [39].

#### 2.2.2. Outcome Variables

The process for confirming fatal and nonfatal stroke cases has been previously described in detail [28,40]. In summary, physician reviewers in the Strong Heart Study (SHS) Morbidity and Mortality Committee reviewed medical charts to identify stroke cases and sent those cases to neurologists for confirmation. SHS neurologists classified all stroke cases in accordance with international diagnostic criteria. Using these criteria, strokes were further defined as definite stroke or possible stroke based on criteria previously described [30,41]. Throughout the follow-up period, participants underwent annual morbidity and mortality surveillance from baseline until 31 December 2021, which includes a thorough review of tribal and Indian Health Service hospital records, death certificates, and direct contact between study personnel, participants, and participants’ families [30]. Among those without prevalent stroke at baseline, we identified incident possible and definite strokes occurring during the follow-up period. In instances of multiple events in the same individual, the date of the initial event was treated as the first stroke date. Cohort-specific median follow-up time for participants from the original cohort and family cohort was 20.46 and 19.34 years, respectively.

### 2.3. Analysis

We used frequencies, percents, means, and standard deviations to describe participants’ demographic characteristics and stroke risk factors. Differences between baseline current, former, and nonsmokers among the combined cohorts, and stratified by original cohort and family cohort, were determined via chi-square tests and ANOVA for categorical and continuous variables, respectively. Means and standard deviations were used to describe smoking duration in years, daily hours exposed to secondhand smoke, daily cigarette count, and packs per year.

#### 2.3.1. Incidence Rates

Person–time stroke incidence rates were calculated for participants from three study centers, combined, and stratified by cohort (original, family, combined), sex (male, female, combined male and female), and age [<55, 55–64, 65]. Stroke included definite or possible, fatal or non-fatal stroke; ischemic and hemorrhagic stroke subtypes were not analyzed separately and were considered together in all analyses. Log-rank tests were used to investigate differences in survival distribution by smoking status (current smokers, former smokers, nonsmokers) and cohort, sex, and age.

#### 2.3.2. Cox Proportional Hazards

To account for relatedness within the family cohort, we used shared frailty Cox proportional hazards models to assess the association of incident stroke with baseline smoking status while controlling for covariates that include age, sex, center, cohort, baseline alcohol consumption status, waist circumference, hypertension, diabetes mellitus (DM), HDL cholesterol (HDL-C), and albuminuria.

The initial model (Model 1) examined the influence of baseline smoking on the time to the first occurrence among those who were stroke-free at baseline. Model 2 added demographic variables (age and sex). Model 3 added modifiable risk factors (baseline alcohol consumption status, diabetes mellitus (DM), hypertension, albuminuria, HDL-C, and waist circumference). Finally, for Model 4, we employed backward stepwise regression to remove statistically insignificant variables (*p*-value > 0.05) from Model 3. Two-way interactions between baseline smoking status and key demographic and cohort variables (age, sex, cohort, and study center) were tested, and no significant interactions were found.

## 3. Results

Among 5847 participants from the original and family cohorts, 39 were excluded due to prevalent stroke (33 original, 6 family) and 6 were excluded due to missing smoking data, yielding a final analytical sample of 5802 participants. This included 3465 participants enrolled in the original cohort in 1989–1991 and 2337 participants enrolled in the family cohort in 2001–2003. In the final sample, 2220 (38.3%) were current baseline smokers. Bivariate analysis of participant characteristics by baseline smoking status and cohort indicated age, sex, and center differed by smoking status (all *p*-values < 0.001, Table 1). Current smokers were younger and more likely to be female or from North and South Dakota areas.

Current smokers enrolled in the original cohort reported a longer duration of smoking and higher secondhand smoke exposure compared to those who reported former smoking (mean 29.47 ± 13.77 years, *n* = 1315 vs. mean 17.99 ± 13.58 years, *n* = 1111) and (mean 5.44 ± 4.83 h/day, *n* = 1313 vs. mean 2.29 ± 3.46 h/day, *n* = 1130), respectively. Current smokers enrolled in the original cohort reported a mean daily cigarette consumption of 12.73 ± 12.67 cigarettes (*n* = 1316) and a mean annual cigarette consumption of 19.71 ± 19.92 packs (*n* = 1305). Similarly, current smokers enrolled in the family cohort reported longer smoking duration and greater secondhand smoke exposure than their former smoking counterparts (mean 19.11 ± 12.35 years, *n* = 890 vs. mean 15.86 ± 11.42 years, *n* = 499, and mean 3.94 ± 5.01 h/day, *n* = 899 vs. mean 2.49 ± 4.39 h/day, *n* = 512), respectively. Current smokers enrolled in the family cohort reported a mean daily cigarette consumption of 8.54 ± 8.10 (*n* = 901) and a mean annual cigarette consumption of 9.43 ± 13.55 packs (*n* = 890).

**Figure 1:**
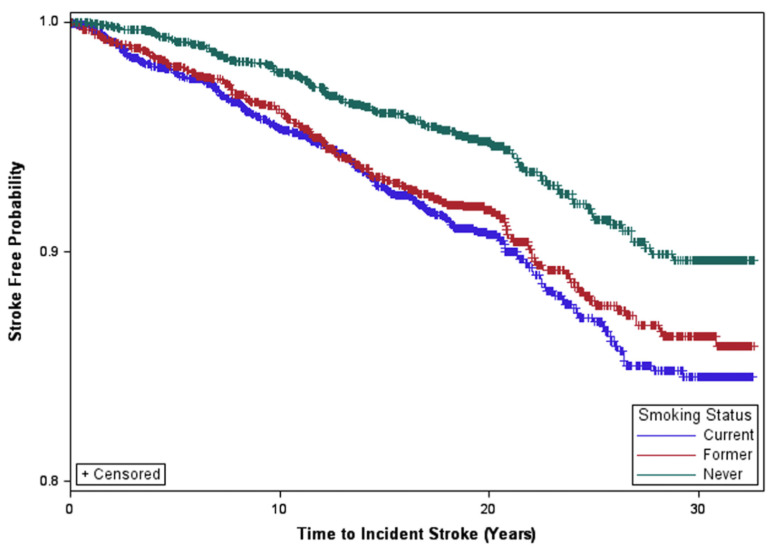
Survival Curve for Time to Incident Stroke by Smoking Status.

Additionally, alcohol consumption, higher waist circumference, hypertension, diabetes mellitus, HDL-C, and albuminuria varied significantly between smoking groups with smokers (current and former) generally exhibiting a higher prevalence of these risk factors. We observed incident stroke in 456 participants (7.9%) during a pooled median follow-up time for the combined cohorts of 19.54 years (range, 0.02–32.62). There were 348 definite strokes and 52 possible strokes recorded for the original cohort and 42 definite strokes and 5 possible strokes recorded for the family cohort. Log-rank tests indicated that stroke incidence rates per 100,000 person-years (Table 2) were higher for current smokers in the combined cohorts and the original cohort overall, and among those aged 65+ in the combined cohorts and the original cohort overall (all *p*-values < 0.001, Supplemental Table S1. Figure 1 illustrates the survival curves for time to incident stroke stratified by smoking status, demonstrating shorter stroke-free survival for current smokers compared to former smokers and nonsmokers.

With the proportional hazards assumption met, shared frailty Cox proportional hazards modeling indicated a shorter time to incident stroke for both current and former smokers compared to nonsmokers (Table 3). The hazard ratios for baseline smoking showed that, compared to nonsmokers, current smokers and former smokers had a 69% (HR = 1.69; 95%CI: 1.32–2.16) and 46% (HR = 1.46; 95%CI: 1.12–1.90) higher risk of stroke, respectively. The addition of demographic variables in Model 2 raised the hazard ratio for current smoking to 1.84 (95%CI: 1.44–2.35). Inclusion of modifiable risk factors (e.g., current drinking at baseline, waist circumference, hypertension, diabetes, HDL-C, and albuminuria) in Model 3 further increased the risk of stroke in current smokers compared to non-smokers (HR = 2.21; 95%CI: 1.71–2.86). After removing non-significant variables from Model 3 via backward stepwise regression to generate Model 4, the hazard ratio for current smoking remained highly significant (HR = 2.23; 95%CI: 1.73–2.88). Current smokers also demonstrated a significantly higher stroke risk compared to former smokers (Table 4), with an overall hazard ratio for baseline current smoking as compared to former smoking of 1.69 (95%CI: 1.34–2.13).

## 4. Discussion

The current study provides a critical update on stroke incidence and risk factors in an underserved population that continues to demonstrate significantly elevated smoking prevalence in spite of national declines in cigarette use. Notably, historical reports suggest that this population had higher-than-national-average smoking rates at each cohort’s enrollment in 1988 and 2001 [42,43]. Incident stroke was higher among smokers and older participants, as well as participants enrolled in the original cohort. Overall, baseline current smokers had a 2.23-fold increased risk of incident stroke as compared to nonsmokers, and a 1.69-fold increased risk of stroke as compared to former smokers, after controlling for covariates. This provides very robust evidence for the benefits of smoking cessation in a demographic with increased smoking and stroke. Previous studies examining stroke incidence among the SHS population have found similarly increased incidence among smokers and older participants [28,44,45]. While the most recent of these used morbidity and mortality surveillance data through 2010, the current study adds another decade of follow-up time (through 2021) and incorporates the younger and demographically distinct family cohort, enrolled approximately 20 years later (1989–1991 vs. 2001–2003).

Of the existing studies exploring stroke incidence in the original cohort, Zhang et al. (2008) [28] found an incidence for all participants of 588 per 100,000 and of 709 per 100,000 for current smokers at enrollment, with a total of 306 (6.8%) incident strokes and follow-up through 2004. More recently, Wang et al. (2017) [44] found an incidence of 567 per 100,000 for all participants, with a total of 297 (8.5%) incident strokes and follow-up through 2010. The current analysis builds on their findings by adding 12 years of data collection and including the family cohort for the first time. We observed an incidence of 613 (533–673) per 100,000 for the original cohort, with a total of 400 (11.5%) incident strokes, and follow-up through 2021.

The follow-up for an aging original cohort population most likely contributed to their higher incidence rates. The family cohort enrolled a younger population (14–93 years) as compared to the original cohort (45–74 years), with similar baseline smoking prevalence (38.1% for the original cohort vs. 38.6% for the family cohort). Given the increased risk of stroke among smokers, we speculate that this effect may be explained by decreased cigarette use over time due to tribal smoking cessation efforts, resulting in lower stroke incidence during the study’s follow-up period, which would have a more pronounced impact on members of the younger family cohort (78.4% ≤ 54 years).

Both baseline current smokers and former smokers had a higher risk of incident stroke compared to non-smokers; however, while the risk for current smokers is comparable to the 2.3-fold increase in risk among SHS smokers reported in 2004, former smokers possessed a markedly decreased risk comparatively [28]. In both the original and family cohorts, those who smoked were more likely to possess other stroke risk factors (i.e., diabetes mellitus, hypertension, increased waist circumference). Adverse health risk factors tend to co-occur, compounding individual detrimental effects [46]. Smoking in particular tends to accompany other risk factors such as low physical activity, poor diet, high alcohol consumption [47]. It is plausible that improvements in managing other modifiable stroke risk factors over time, such as improved diabetes and hypertension control, have mitigated some of the stroke risk attributable to smoking. The Global Burden of Diseases, Injuries, and Risk Factors Study (GBD) found that 87% of stroke risk could be attributed to modifiable risk factors (e.g., hypertension, obesity, hyperglycemia, hyperlipidemia, and renal dysfunction), and 46% could be attributed to behaviorial risk factors (e.g., smoking, sedentary lifestyle, unhealthy diet) [17]. Additionally, smoking can cause hypertension by promoting endothelial dysfunction, oxidative stress, sympathetic activation, and accelerated vascular stiffening [48]. However, smoking is also a direct and independent risk factor for both ischemic and hemorrhagic stroke, as shown by several large meta-analyses and Mendelian randomization studies even after adjusting for hypertension and vascular risk factors [49]. Although we adjusted for these factors in multivariate analysis, there may be some residual confounding.

Baseline current smokers had a 69% higher risk of stroke as compared to former smokers, providing very compelling evidence for the stroke-related benefits of smoking cessation. Smoking rates for the American Indian population remain high nationally and provide a ripe opportunity for targeted intervention. Quitting smoking at any age lowers mortality from stroke and other smoking-related diseases [50]. By decreasing smoking rates in this population, a concomitant decrease in other stroke risk factors is likely to occur given their disproportionate presence in smoking populations. Only a small number of studies examined factors that contributed to American Indians’ smoking. They typically incorporate individual counseling, validated cessation tools, and collaboration with tribal communities. Common obstacles include lack of follow-up due to geographical isolation, distrust, and poor American Indian representation among researchers [51]. These barriers may be obviated by cultural and strength-based interventions [52]. Furthermore, successful intervention studies likely depend on dedicated support from funders, researchers, and trust-based collaboration with local communities. These results would support additional large-scale public health initiatives and inform high-impact targeted interventions for widely underserved populations [52].

### Limitations

While our findings provide valuable insight into the relationship between smoking and stroke over time in the American Indian population, the current study has several important limitations. Stroke incidence and risk were not examined by stroke type (i.e., ischemic, hemorrhagic). Although stroke subtypes were adjudicated by SHS neurologists using standardized criteria, subtype-specific analyses were not performed due to concerns regarding limited statistical power, particularly after stratification by smoking status, cohort, age, and sex. While ischemic and hemorrhagic strokes share several major risk factors, including smoking and hypertension [4], their underlying pathophysiology and relative risk profiles differ. As a result, our findings should be interpreted as reflecting overall stroke risk rather than subtype-specific associations. Additionally, while the relatedness of participants in the family cohort was addressed in shared frailty Cox proportional hazards models, incidence was not similarly adjusted. The generalizability of our study should be considered, as attitudes towards smoking can vary significantly between communities. Furthermore, smoking status was self-reported, which could introduce bias, and only baseline smoking data were included in the analysis.

Despite these limitations, the study has several notable strengths. As a prospective cohort study, it establishes a temporal sequence between smoking and stroke. The inclusion of three sites for data collection enhances the generalizability of the findings to American Indian populations or populations with high cardiometabolic risk factors. The Strong Heart Study (SHS) was specifically designed to fill a research gap in addressing cardiovascular disease (CVD) in American Indians. Moreover, the meticulous morbidity and mortality surveillance (M&M) process of the SHS ensures a high level of accuracy in classifying incident stroke, minimizing the risk of misclassification.

## 5. Conclusions

Smoking remains a key risk factor for stroke in American Indians. The current study extends our prior research by widening the scope of analysis to include a broader range of participant ages and a more extended follow-up period in two cohorts of American Indians. While American Indians continue to have higher smoking rates and stroke risk, an increased risk of stroke in current smokers as compared to former smokers strongly supports the benefits of smoking cessation and emphasizes the need for culturally tailored smoking cessation interventions and ongoing stroke surveillance.

## Figures and Tables

**Table 1:** Participant Characteristics by Baseline Smoking Status and Cohort.

	Original Cohort and Family Cohort *n* (%)									
	All *n* = 5802			Current Smoker *n* = 2220		Former Smoker *n* = 1651		Nonsmoker *n* = 1931		*p*
Age										<0.001
<55	3830 (66.0%)			1576 (71.0%)		972 (58.9%)		1282 (66.4%)		
55 to 64	1263 (21.8%)			455 (20.5%)		415 (25.1%)		393 (20.4%)		
65+	709 (12.2%)			189 (8.5%)		264 (16.0%)		256 (13.3%)		
Sex										<0.001
Male	2399 (41.3%)			994 (44.8%)		863 (52.3%)		617 (32.0%)		
Female	3403 (58.7%)			1226 (55.2%)		788 (47.7%)		1314 (68.0%)		
Center										<0.001
Arizona	742 (23.8%)			153 (6.9%)		247 (15.0%)		341 (17.7%)		
Oklahoma	2479 (42.7%)			857 (38.6%)		744 (45.1%)		878 (45.5%)		
South Dakota	2582 (44.5%)			1210 (54.4%)		660 (44.0%)		712 (36.9%)		
Current alcohol consumption										<0.001
Yes	2949 (50.8%)			1421 (64.0%)		944 (57.2%)		1110 (57.5%)		
No	2853 (49.2%)			1528 (42.7%)		707 (42.8%)		821 (42.5%)		
Waist Circumference (inches) M (SD)	40.56 (6.39)			39.72 (6.25)		41.73 (5.87)		40.52 (6.81)		<0.001
Hypertension										<0.001
Yes	1887 (32.5%)			582 (26.2%)		643 (38.9%)		662 (34.3%)		
No	3915 (67.5%)			1638 (73.8%)		1008 (61.1%)		1269 (65.7%)		
Diabetes Mellitus										<0.001
Yes	1673 (28.8%)			542 (24.4%)		597 (36.2%)		534 (27.7%)		
No	4129 (71.2%)			1678 (75.6%)		1054 (63.8%)		1397 (72.3%)		
HDL-C										<0.001
≥1.554 (60 mg/dL)	1061 (18.6%)			392 (17.9%)		262 (16.2%)		407 (21.4%)		
1.036–1.528 (40–59 mg/dL)	3029 (53.1%)			1119 (51.15)		852 (52.7%)		1058 (55.7%)		
<1.036	1616 (28.35)			678 (31.0%)		504 (31.1%)		504 (22.9%)		
Albuminuria										<0.001
Normal	4540 (80.1%)			1810 (83.3%)		1226 (76.2%)		1504 (79.6%)		
Micro	799 (14.1%)			267 (12.3%)		249 (15.5%)		283 (15.0%)		
Macro	331 (5.8%)			95 (4.4%)		133 (8.3%)		103 (5.4%)		
	**Original Cohort *n* (%)**					**Family Cohort *n* (%)**				
	**All** **(*n* = 3465)**	**Current Smoker *n* = 1319**	**Former Smoker** ***n* = 1138**	**Nonsmoker** ***n* = 1008**	***p***	**All** ***n* = 2337**	**Current Smoker** ***n* = 901**	**Former Smoker** ***n* = 513**	**Nonsmoker** ***n* = 923**	***p***
Age					<0.001					<0.001
<55	1684 (48.6%)	727 (55.1%)	530 (46.6%)	427 (42.4%)		2146 (91.8%)	849 (94.2%)	442 (86.2%)	855 (92.6%)	
55 to 64	1148 (33.1%)	413 (31.3%)	373 (32.8%)	362 (35.9%)		115 (4.9%)	42 (4.7%)	42 (8.2%)	31 (3.4%)	
65 +	633 (18.3%)	179 (13.6%)	235 (20.7%)	219 (21.7%)		76 (3.3%)	10 (1.1%)	29 (5.7%)	37 (4.0%)	
Sex					<0.001					0.154
Male	1430 (41.3%)	614 (46.6%)	561 (49.3%)	255 (25.3%)		969 (41.5%)	380 (42.2%)	227 (44.2%)	362 (39.2%)	
Female	2035 (58.75)	705 (53.4%)	577 (50.7%)	753 (74.7%)		1368 (58.5%)	521 (57.8%)	286 (55.8%)	561 (60.8%)	
Center					<0.001					<0.001
Arizona	462 (13.3%)	79 (6.0%)	178 (15.6%)	205 (20.3%)		279 (11.9%)	74 (8.2%)	69 (13.5%)	136 (14.7%)	
Oklahoma	1497 (43.2%)	508 (38.5%)	527 (46.3%)	462 (45.8%)		982 (42.0%)	349 (38.7%)	217 (42.3%)	416 (45.1%)	
South Dakota	1506 (43.5%)	732 (55.5%)	433 (38.0%)	341 (33.8%)		1076 (46.0%)	478 (53.1%)	227 (44.2%)	371 (40.2%)	
Current alcohol consumption					<0.001					<0.001
Yes	1452 (41.9%)	730 (55.3%)	410 (36.0%)	312 (31.0%)		1497 (64.1%)	691 (76.7%)	297 (57.9%)	509 (55.1%)	
No	2013 (58.15)	589 (44.7%)	728 (64.0%)	696 (69.0%)		840 (35.9%)	210 (23.3%)	216 (42.1%)	414 (44.9%)	
Waist Circumference (inches) M (SD)	40.95 (5.59)	39.71 (5.59)	41.84 (5.22)	41.56 (5.69)	<0.001	39.98 (7.38)	39.74 (7.11)	41.47 (7.10)	39.39 (7.69)	<0.001
Hypertension					<0.001					<0.001
Yes	1289 (37.2%)	379 (28.7%)	475 (41.7%)	435 (43.2%)		598 (25.6%)	203 (22.5%)	168 (32.7%)	227 (24.6%)	
No	2176 (62.8%)	940 (71.3%)	663 (58.3%)	573 (56.8%)		1739 (74.4%)	698 (77.5%)	345 (67.3%)	696 (75.4%)	
Diabetes Mellitus					<0.001					<0.001
Yes	1338 (38.6%)	418 (31.7%)	499 (43.8%)	421 (41.8%)		335 (14.3%)	124 (13.8%)	98 (19.1%)	113 (12.2%)	
No	2127 (61.4%)	901 (68.3%)	639 (56.2%)	587 (58.2%)		2002 (85.7%)	777 (86.2%)	415 (80.9%)	810 (87.8%)	
HDL-C					<0.001					0.148
≥1.554 (60 mg/dL)	509 (15.0%)	185 (14.2%)	148 (13.2%)	176 (17.9%)		552 (23.6%)	207 (23.0%)	114 (22.2%)	231 (25.0%)	
1.036–1.528 (40–59 mg/dL)	1727 (50.7%)	629 (48.3%)	560 (50.1%)	538 (54.7%)		1302 (55.7%)	490 (54.4%)	292 (56.9%)	520 (56.3%)	
<1.036	1168 (34.3%)	489 (37.5%)	409 (36.6%)	270 (27.4%)		448 (19.2%)	189 (21.0%)	95 (18.5%)	164 (17.8%)	
Albuminuria					<0.001					0.019
Normal	2553 (75.8%)	1024 (79.7%)	809 (72.9%)	720 (73.8%)		1987 (86.4%)	786 (88.6%)	417 (83.6%)	784 (85.8%)	
Micro	547 (16.2%)	182 (14.2%)	189 (17.0%)	176 (18.0%)		252 (11.0%)	85 (9.6%)	60 (12.0%)	107 (11.7%)	
Macro	270 (8.0%)	79 (6.1%)	111 (10.0%)	80 (8.2%)		61 (2.7%)	16 (1.8%)	22 (4.4%)	23 (2.5%)	

**Table 2:** Stroke Incidence Rates per 100,000 Person-Years in the Original and Family Cohort.

Original and Family Cohort												
	All Participants			Current Smokers			Former Smokers			Nonsmokers		
	Stroke Events	Person-Years	Rate (95% CI)	Stroke Events	Person-Years	Rate (95% CI)	Stroke Events	Person-Years	Rate (95% CI)	Stroke Events	Person-Years	Rate (95% CI)
Male												
Total Participants	178	42,315	421 (359–482)	83	16,873	492 (386–598)	65	14,355	453 (343–563)	30	11,086	271 (174–367)
<55	20	16,820	119 (67–171)	9	6791	133 (46–219)	5	3812	132 (16–246)	6	6217	97 (19–174)
55–64	45	10,500	429 (303–554)	27	4697	575 (358–792)	12	3758	319 (139–500)	6	2045	293 (59–528)
65+	113	15,332	737 (601–873)	47	5558	846 (604–1087)	48	6881	698 (500–895)	18	2894	622 (335–909)
Female												
Total Participants	278	65,412	425 (375–475)	119	22,783	522 (428–616)	79	16,708	473 (369–577)	80	25,921	309 (241–376)
<55	19	22,914	83 (46–120)	11	9062	121 (50–193)	6	4745	126 (25–228)	2	9107	22 (−8–52)
55–64	56	16,110	348 (257–439)	28	6040	464 (292–635)	19	4507	422 (232–611)	9	5563	162 (56–267)
65+	203	26,944	753 (650–857)	80	7992	1001 (782–1220)	54	7618	709 (520–898)	69	11,334	609 (465–752)
Males and females												
Total Participants	456	107,727	423 (384–462)	202	39,656	509 (439–580)	144	31,063	464 (388–539)	110	37,008	297 (242–353)
<55	39	39,734	98 (67–129)	20	15,853	126 (71–181)	11	8557	129 (53–205)	8	15,324	52 (16–88)
55–64	101	26,610	380 (306–454)	55	10,737	512 (377–648)	31	8265	375 (243–507)	15	7609	197 (97–297)
65+	316	42,276	747 (665–830)	127	13,550	937 (774–1100)	102	14,498	704 (567–840)	87	14,228	611 (483–740)
**Original Cohort**												
	**All Participants**			**Current Smokers**			**Former Smokers**			**Nonsmokers**		
	**Stroke Events**	**Person-Years**	**Rate (95% CI)**	**Stroke Events**	**Person-Years**	**Rate (95% CI)**	**Stroke Events**	**Person-Years**	**Rate (95% CI)**	**Stroke Events**	**Person-Years**	**Rate (95% CI)**
Male												
Total Participants	153	25,008	612 (515–709)	74	10,128	731 (564–897)	56	10,416	538 (397–678)	23	4464	515 (305–726)
<55	9	3534	255 (88–421)	5	1690	296 (37–555)	3	1303	230 (−30–491)	1	583	171 (−165–508)
55–64	39	7862	496 (340–652)	24	3543	677 (406–948)	10	2993	334 (127–541)	5	1325	377 (47–708)
65+	105	13,870	757 (612–902)	45	5086	885 (626–1143)	43	6197	694 (487–901)	17	2587	657 (345–969)
Female												
Total Participants	247	40,231	614 (537–691)	104	13,314	781 (631–931)	71	11,647	619 (475–763)	72	15,450	466 (358–574)
<55	10	4643	215 (82–349)	7	1977	354 (92–616)	2	1288	155 (−60–370)	1	1397	72 (−69–212)
55–64	45	11,568	389 (275–503)	21	4350	483 (276–689)	17	3295	516 (271–761)	7	3924	178 (46–311)
65+	192	24,484	784 (673–895)	76	7282	1044 (809–1278)	52	7014	741 (540–943)	64	10,188	628 (474–782)
Males and females												
Total Participants	400	65,962	613 (553–673)	178	23,442	759 (648–871)	127	21,883	580 (479–681)	95	19,914	477 (381–573)
<55	19	8177	232 (128–337)	12	3605	333 (145–521)	5	2592	193 (24–362)	2	1980	101 (−39–241)
55–64	84	19,430	432 (340–525)	45	7893	570 (404–737)	27	6288	429 (267–591)	12	5250	229 (99–358)
65+	297	38,355	774 (686–862)	121	12,368	978 (804–1153)	95	13,211	719 (574–864)	81	12,776	634 (496–772)
**Family Cohort**												
	**All Participants**			**Current Smokers**			**Former Smokers**			**Nonsmokers**		
	**Stroke Events**	**Person-Years**	**Rate (95% CI)**	**Stroke Events**	**Person-Years**	**Rate (95% CI)**	**Stroke Events**	**Person-Years**	**Rate (95% CI)**	**Stroke Events**	**Person-Years**	**Rate (95% CI)**
Male												
Total Participants	25	17,307	144 (88–201)	9	6745	133 (46–221)	9	3939	228 (79–378)	7	6623	106 (27–184)
<55	11	13,286	83 (34–132)	4	5143	78 (2–154)	2	2509	80 (−31–190)	5	5634	89 (11–167)
55–64	6	2638	227 (45–409)	3	1153	260 (−34–554)	2	765	185 (−101–624)	1	720	139 (−133–411)
65+	8	1461	547 (168–927)	2	471	424 (−164–1012)	5	684	731 (90–1372)	1	306	327 (−314–967)
Female												
Total Participants	31	25,182	123 (80–166)	15	9469	158 (78–239)	8	5241	153 (47–258)	8	10,471	76 (24–129)
<55	9	18,271	49 (17–81)	4	7104	56 (1–111)	4	3457	116 (2–229)	1	7710	13 (−13–38)
55–64	11	4542	242 (99–385)	7	1691	414 (107–721)	2	1212	165 (−64–394)	2	1639	122 (−47–291)
65+	11	2460	447 (183–711)	4	710	563 (11–1115)	2	604	331 (−128–791)	5	1146	436 (54–819)
Males and females												
Total Participants	56	42,489	132 (97–166)	24	16,214	148 (89–207)	17	9180	185 (97–273)	15	17,094	88 (43–132)
<55	20	31,557	63 (36–91)	8	12,248	65 (20–111)	6	5965	101 (20–181)	6	13,344	45 (9–81)
55–64	17	7180	237 (124–349)	10	2844	352 (134–569)	4	1977	202 (4–401)	3	2359	127 (−17–271)
65+	19	3921	485 (267–702)	6	1182	508 (101–914)	7	1287	544 (141–947)	6	1452	413 (83–744)

**Table 3:** Cox Proportional Hazards Model: Time to First Fatal or Non-Fatal Stroke (Definite or Possible) Comparing Current and Former Smoking vs. Nonsmoking.

	Hazard Ratios (95% CI)			
	Model 1	Model 2	Model 3	Model 4
Current smoking				
Current Smoker (vs. nonsmoker)	1.69 (1.32–2.16)	1.84 (1.44–2.35)	2.21 (1.71–2.86)	2.23 (1.73–2.88)
Former Smoker (vs. nonsmoker)	1.46 (1.12–1.90)	1.30 (1.01–1.68)	1.36 (1.05–1.77)	1.36 (1.04–1.76)
Age, y		1.07 (1.06–1.08)	1.06 (1.05–1.07)	1.06 (1.05–1.07)
Sex (male vs. female)		1.02 (0.84–1.24)	1.06 (0.86–1.30)	
Site				
Oklahoma (vs. Arizona)		1.15 (0.80–1.65)	1.40 (0.96–2.05)	1.42 (0.97–2.07)
Dakotas (vs. Arizona)		1.67 (1.16–2.39)	2.33 (1.60–3.41)	2.33 (1.60–3.40)
Cohort (Original vs. Family)		1.67 (1.23–2.27)	1.71 (1.25–2.35)	1.75 (1.28–2.40)
Current alcohol use (vs. no current alcohol use)			0.74 (0.60–0.92)	0.74 (0.59–0.91)
Waist circumference, in			1.00 (0.98–1.02)	
Hypertension (vs. no hypertension)			1.51 (1.22–1.86)	1.50 (1.22–1.84)
Diabetes (vs. normal glucose tolerance)			1.64 (1.32–2.04)	1.65 (1.23–2.04)
High density lipoprotein cholesterol, mg/dL			1.00 (0.99–1.01)	
Albuminuria (vs. normal)			1.59 (1.26–2.00)	1.59 (1.26–2.01)

**Table 4:** Cox Proportional Hazards Model: Time to First Fatal or Non-Fatal Stroke (Definite or Possible) Comparing Current Smoking vs. Former Smoking.

	Hazard Ratios (95% CI)			
	Model 1	Model 2	Model 3	Model 4
Current smoking (vs. former smoking)	1.15 (0.91–1.44)	1.43 (1.14–1.78)	1.64 (1.30–2.08)	1.69 (1.34–2.13)
Age, y		1.07 (1.06–1.09)	1.06 (1.05–1.08)	1.06 (1.05–1.08)
Sex (male vs. female)		0.95 (0.77–1.18)	0.99 (0.79–1.26)	
Site				
Oklahoma (vs. Arizona)		1.06 (0.68–1.64)	1.26 (0.80–2.00)	1.30 (0.82–2.05)
Dakotas (vs. Arizona)		1.42 (0.92–2.19)	1.98 (1.25–3.12)	1.99 (1.26–3.14)
Cohort (Original vs. Family)		1.65 (1.15–2.36)	1.79 (1.23–2.60)	1.81 (1.25–2.63)
Current alcohol use (vs. no current alcohol use)			0.67 (0.53–0.86)	0.68 (0.54–0.87)
Waist circumference, in			0.99 (0.97–1.01)	
Hypertension (vs. no hypertension)			1.51 (1.19–1.92)	1.48 (1.17–1.88)
Diabetes (vs. normal glucose tolerance)			1.78 (1.38–2.30)	1.72 (1.35–2.19)
High density lipoprotein cholesterol, mg/dL			1.00 (0.99–1.01)	
Albuminuria (vs. normal)			1.63 (1.25–2.14)	1.64 (1.25–2.14)
Current smoking (vs. former smoking)	1.15 (0.91–1.44)	1.43 (1.14–1.78)	1.64 (1.30–2.08)	1.69 (1.34–2.13)
Age, y		1.07 (1.06–1.09)	1.06 (1.05–1.08)	1.06 (1.05–1.08)

## Data Availability

Due to privacy agreements with participating tribal communities, access to study data is restricted. Qualified researchers with human subject confidentiality training can request access to datasets through the SHS Coordinating Center at https://strongheartstudy.org/ (accessed on 1 December 2025).

## Supplementary Materials

The following supporting information can be downloaded at: https://www.mdpi.com/article/10.3390/jcm15020431/s1, Table S1: Differences in incidence rates by smoking status (current smokers, former smokers, nonsmokers).

## Abbreviations

The following abbreviations are used in this manuscript:

SHS	Strong Heart Study
HDL-C	High-Density Lipoprotein
NHIS	National Health Interview Survey
BRFSS	Behavioral Risk Factor Surveillance System
NH	Non-Hispanic
DM	Diabetes Melltius
JNC	Joint National Committee
M&M	Morbidity and Mortality
CVD	Cardiovascular Disease
