# Supplementary material for: Stroke Incidence and Smoking in American Indians: An Update from the Strong Heart Study
Source: J Clin Med. 2026 Jan 6;15(2):431. doi: 10.3390/jcm15020431 (PMC12841873; doi:10.3390/jcm15020431)
Supplement: Supplementary file 1 [file jcm-15-00431-s001.zip › jcm-3995898-supplementary.pdf]

Supplemental Table S1. *Differences in incidence rates by smoking status (current smokers, former smokers, nonsmokers)*

	Original and Family Cohorts		Original Cohort		Family Cohort	
	χ^2	p	χ^2	p	χ^2	p
**Male**						
Total Participants	**7.7**	**0.02**	4	0.100	2.7	0.300
< 55	0.5	0.800	0.3	0.900	0	1.000
55-64	4.3	0.100	4.4	0.100	0.3	0.800
65+	2	0.400	2.2	0.300	0.5	0.800
**Female**						
Total Participants	**14.6**	**>.001**	**11.9**	**0.003**	3.2	0.200
< 55	**6.5**	**0.040**	3.3	0.200	5.3	0.070
55-64	**8.5**	**0.010**	**6.7**	**0.030**	3.3	0.200
65+	**14.1**	**>.001**	**13.6**	**0.001**	0.6	0.800
**Males and females**						
Total Participants	**22**	**>.001**	**14.9**	**>.001**	4.8	0.090
< 55	5.2	0.080	3.2	0.200	2.2	0.300
55-64	**11.7**	**0.003**	**8.5**	**0.010**	2.9	0.200
65+	**15.2**	**>.001**	**15**	**>.001**	0.3	0.900
